# Adding Consolidation Capecitabine to Neoadjuvant Chemoradiotherapy for Locally Advanced Rectal Cancer: A Propensity-Matched Comparative Study

**DOI:** 10.3389/fsurg.2021.770767

**Published:** 2022-01-27

**Authors:** Yifang Fang, Chengmin Sheng, Feng Ding, Weijie Zhao, Guoxian Guan, Xing Liu

**Affiliations:** ^1^Department of Colorectal Surgery, The First Affiliated Hospital of Fujian Medical University, Fuzhou, China; ^2^Fuzhou Medical College of Nanchang University, Fuzhou, China

**Keywords:** rectal neoplasm, neoadjuvant chemoradiotherapy, capecitabine, prognosis, propensity score matched analysis

## Abstract

**Aim:**

To determine whether adding consolidation capecitabine chemotherapy without lengthening the waiting period influences pathological complete response (pCR) and short-term outcome of locally advanced rectal cancer (LARC) receiving neoadjuvant chemoradiotherapy (NCRT).

**Method:**

Totally, 545 LARC who received NCRT and radical resection between 2010 and 2018 were enrolled. Short-term outcome and pCR rate were compared between patients with and without additional consolidation capecitabine. Logistic analysis was performed to identify predictors of pCR.

**Results:**

After propensity score matching, 229 patients were matched in both NCRT and NCRT-Cape groups. Postoperative morbidity was comparable between groups except for operation time, which is lower in the NCRT group (213.2 ± 67.4 vs. 227.9 ± 70.5, *p* = 0.025). Two groups achieved similar pCR rates (21.8 vs. 22.7%, *p* = 1.000). Tumor size (OR = 0.439, *p* < 0.001), time interval between NCRT and surgery (OR = 1.241, *p* = 0.003), and post-NCRT carcinoembryonic antigen (OR = 0.880, *p* = 0.008) were significantly correlated with pCR in patients with LARC. A predictive nomogram was constructed with a C-index of 0.787 and 0.741 on internal and external validation.

**Conclusion:**

Adding consolidation capecitabine chemotherapy without lengthening CRT-to-surgery interval in LARC patients after NCRT does not seem to impact pCR or short-term outcome. A predictive nomogram for pCR was successful, and it could support treatment decision-making.

## Introduction

Neoadjuvant chemoradiotherapy (NCRT) and radical surgery have become the standard treatment for locally advanced rectal cancer (LARC) (1). The benefits of this multimodal treatment have been well-documented, namely, tumor downsizing and downstaging, increased radical resection rate, and better local tumor control (2–4). Approximately 10–30% of LARC patients following NCRT will develop a pathological complete response (pCR), together with a low recurrence rate (5–7).

Given the superior oncological outcome, organ preservation with a “watch and wait” strategy or local excision, has been proposed to patients achieving pCR to improve the quality of life and anal sphincter preserving rate. Consequently, increasing pCR rate has become a primary endpoint of clinical trials, which might increase patients with LARC who could potentially benefit from organ-preservation strategies. Many strategies have been adopted to maximize the pCR rate, namely, dose-escalated radiation (8), intensified neoadjuvant treatment [induction (9) or consolidation chemotherapy (1, 8, 10–16)], and lengthening the CRT-to-surgery interval (17).

Standard NCRT protocol using a continuous infusional 5-fluorouracil (5-Fu) for radiation sensitization has been shown to achieve tumor downstaging, but no improved overall survival. Growing evidence has demonstrated that the addition of oxaliplatin to NRT acquires equivalent oncological outcomes when compared to fluoropyrimidine-based CRT, but increases toxicities and cost (18–22). The inconvenience of using an intravenous continuous infusion of 5-FU resulted in the development of an oral fluoropyrimidine, capecitabine. A meta-analysis (23) has demonstrated equivalent efficacy of capecitabine and infusional 5-Fu in the neoadjuvant setting, suggesting capecitabine to be an alternative to 5-Fu-based CRT for LARC.

In this article, we aimed to determine whether adding 2 cycles of consolidation capecitabine without lengthening CRT-to-surgery interval influences pCR rate and short-term outcome of LARC patients after NCRT. In addition, we sought to identify post-CRT determinants for pCR, and to construct a nomogram that might be helpful during organ preservation strategy decision-making.

## Patients and Method

### Patient Eligibility

We performed a retrospective study based on propensity score matching. Between October 2010 and January 2018, patients with LARC who underwent curative resection and received capecitabine-based NCRT from our database. Patient inclusion criteria were: (1) clinical stage II or III (cT3/4 or cN1/2) disease; (2) pathologically proven rectal adenocarcinomas; and (3) tumors distance <12 cm from the anal verge. Exclusion criteria included: (1) neoadjuvant chemotherapy regimen, namely, oxaliplatin, irinotecan, or molecular targeted agents; (2) previous or concurrent malignancy; (3) emergency or palliative resection; and (4) transanal local excision. This study was approved by the Institutional Review Board of Fujian Medical University Union Hospital (2013051).

### Treatment Protocol and Follow-Up

**Figure 1:**
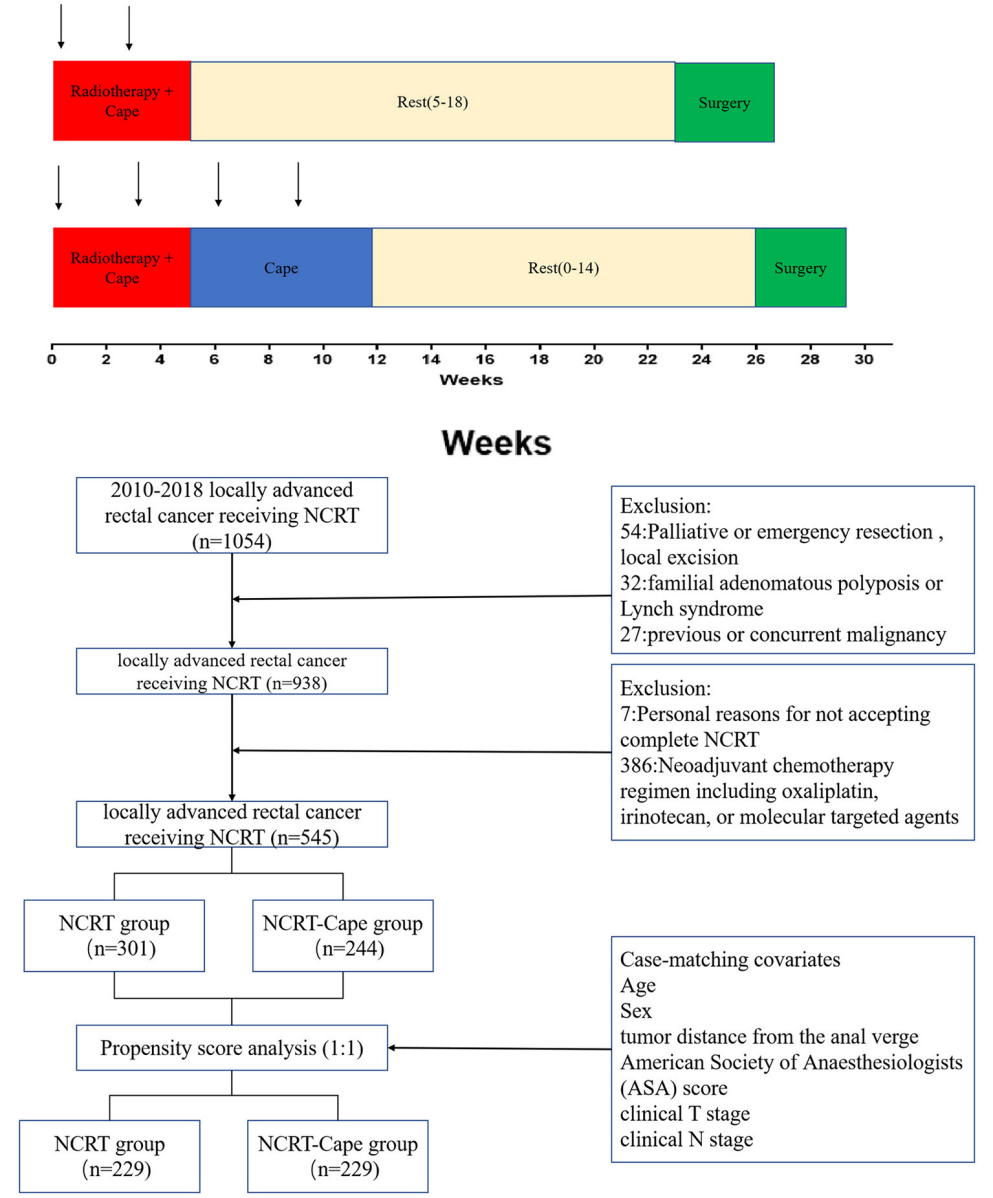
The protocol of the neoadjuvant chemoradiotherapy in the two groups. NCRT, neoadjuvant chemoradiotherapy; NCRT-Cape, neoadjuvant chemoradiotherapy, and additional capecitabine chemotherapy.

Patient assessments were performed at baseline for tumor staging using a digital rectal examination, colonoscopy, chest radiography, abdominopelvic MRI, and/or transrectal ultrasound. Preoperative radiotherapy consisted of 45 Gy to the pelvis for 5 weeks (180 cGy/25 fractions) and a tumor boost of 5.4 Gy. Concomitant chemotherapy was administered with oral capecitabine (825 mg/m^2^ two times daily from day 1 to day 14 per cycle, a total of two cycles during the pre-operative radiotherapy). The treatment decision whether or not to add two-cycle consolidation capecitabine chemotherapy to NCRT was based on the disease stage. Surgery was carried out 6–20 weeks after the completion of radiation. Surgical techniques, namely, total mesorectal excision and high ligation of the inferior mesenteric artery, were routinely performed. After 3–4 weeks from surgery, adjuvant chemotherapy (FOLFOX or CapeOX) was considered for patients for 6 months. The treatment schema of our study is presented in Figure 1.

Patients were followed four times in the first 3 years, then twice for the next 2 years, and annually thereafter. Patient follow-up lasted until death or the cut-off date of October 31, 2018.

### Definitions

Tumor distance from the anal verge was estimated by digital rectal examination, pre-operative MRI evaluation, and intraoperative findings during the operation. Tumor response to NCRT was graded according to Rectal Cancer Tumor Regression Grade (TRG) method (24). pCR was defined as no viable tumor cells in the primary site or the lymph nodes. Postoperative morbidity was classified according to the Clavien–Dindo classification (25).

### Statistical Analysis

To minimize group differences, we performed a 1:1 propensity score matching analysis by using R Version 3.5.1 (Vienna, Austria). Statistical analyses were performed using SPSS version 23.0 (SPSS INC., Chicago, IL, USA). Categorical variables were presented as numbers and compared using the chi-square test or Fisher's exact test as appropriate. Continuous variables were expressed as means ± SD and analyzed using Student's *t*-test. The logistic regression model was used to identify independent predictors for pCR, and a predictive nomogram was developed by the R project. To validate the results, patients were randomly divided into training (*n* = 420) and validation (*n* = 125) cohorts by using SPSS. The nomogram went through internal and external validation. *p* < 0.05 was considered statistically significant.

## Results

### Patient Characteristics

The baseline features of patients with LARC are presented in Table 1. Totally 545 patients with LARC were included. After propensity score matching, 229 patients receiving standard NCRT (NCRT group) and 229 patients treated with NCRT and additional 2 cycles of consolidation capecitabine chemotherapy (NCRT-Cape group) were matched. After matching, between-group baseline characteristics were well-balanced, such as age, gender, American Society of Anesthesiologists grade, interval time between NCRT and surgery, distance from the anal verge, clinical T and N stage.

**Table 1:** Patient characteristics in patients with LARC after NCRT.

**Characteristics**	**Unmatched patients**			**Propensity-matched patients**		
	**NCRT (***n*** = 301)**	**NCRT-Cape (***n*** = 244)**	***p*** **-value**	**NCRT (***n*** = 229)**	**NCRT-Cape (***n*** = 229)**	***p*** **-value**
Sex (%)			0.857			1.000
Male	193 (64.1)	159 (65.2)		148 (64.6)	148 (58.4)	
Female	108 (35.9)	85 (34.8)		81 (35.4)	81 (35.4)	
Age (years)	57.4.0 ± 11.4	56.0 ± 10.7	0.131	57.3 ± 11.4	56.3 ± 10.4	0.348
ASA score (%)			0.842			0.922
1	214 (71.1)	179 (73.4)		167 (72.9)	169 (73.8)	
2	83 (27.6)	62 (25.4)		58 (25.3)	59 (24.9)	
3	4 (1.3)	3 (1.2)		4 (1.7)	3 (1.3)	
Distance from the anal verge (cm)	6.1 ± 2.3	6.7 ± 2.5	**0.002**	6.3 ± 2.5	6.4 ± 2.3	0.656
Time interval between CRT and surgery (weeks)	9.5 ± 1.5	9.3 ± 1.6	0.311	9.5 ± 1.5	9.5 ± 2.6	0.925
Histopathology (%)			0.656			0.248
pCR	64 (21.3)	53 (21.3)		154 (98.1)	157 (100.0)	
Adenocarcinoma	219 (72.8)	173 (70.9)		154 (98.1)	157 (100.0)	
Signet ring cell carcinoma	17 (5.6)	15 (6.1)		3 (1.9)	0 (0.0)	
Mucinous adenocarcinoma	1 (0.3)	3 (1.2)		17 (5.6)	17 (5.6)	
Tumor size	2.6 ± 1.3	2.5 ± 1.3	0.699	2.5 ± 1.2	2.5 ± 1.2	0.485
Clinical T stage (%)			0.063			0.070
T2 + 3	128 (42.5)	84 (34.3)		102 (44.5)	82 (35.8)	
T4	173 (57.5)	160 (65.6)		127 (55.5)	147 (64.2)	
Clinical N stage (%)			0.320			0.446
N0	34 (11.3)	21 (8.6)		27 (11.8)	21 (3.0)	
N+	267 (88.7)	223 (91.4)		202 (88.2)	208 (90.8)	

*LARC, locally advanced rectal cancer; NCRT, neoadjuvant chemoradiotherapy; NCRT-Cape, neoadjuvant chemoradiotherapy and additional capecitabine chemotherapy; ASA, American society of anesthesiologists; CRT, chemoradiotherapy. Bold values indicate of statistical significance*.

### Perioperative Outcomes

Surgical results are listed in Table 2. Estimated blood loss, surgical approach, and preserve organ rate were comparable between the two groups (Table 2). The operation time in the NCRT group was significantly lower than that of the NCRT-Cape group (213.2 ± 67.4 vs. 227.9 ± 70.5, *p* = 0.025), compared to. Postoperative morbidity was similar between two groups (14.4 vs. 17.5%, *p* = 0.444). No group difference was observed in post-operative hospital stay and 30 days readmission (*p* = 0.183, *p* = 1.000, respectively). Complication severity was similar in the two groups. Similarly, no significant difference was observed in peri-NCRT complications between groups (*p* = 0.917). No re-operation was found in either group. Likewise, no perioperative mortality occurred in the two groups.

**Table 2:** Operative and post-operative outcomes in patients with LARC after NCRT.

**Characteristics**	**NCRT (***n*** = 229)**	**NCRT-Cape (***n*** = 229)**	***p*** **-value**
Operative time (min)	213.2 ± 67.4	227.9 ± 70.5	**0.025**
Estimated blood loss (ml)	76.5 ± 88.8	79.2 ± 81.9	0.417
Surgery approach			0.516
Laparoscopic	158 (69.0)	155 (67.6)	
Open	53 (23.1)	49 (21.4)	
Robotic	18 (7.9)	25 (10.9)	
Post-operative hospital stay (days)	8.3 ± 5.5	9.0 ± 6.3	0.183
Post-operative complications	33 (14.4)	40 (17.5)	0.444
30 days readmission	1 (0.4)	1 (0.4)	1.000
Peri-CRT complications*	64 (27.9)	62 (27.1)	0.917
Major	7 (3.1)	1 (0.4)	0.068
Sphincter-saving procedure	203 (88.6)	204 (89.1)	1.000
Lymph nodes retrieved	12.4 ± 7.7	12.5 ± 6.4	0.832
Metastatic lymph nodes	0.5 ± 1.3	0.5 ± 1.5	0.840
CRM involvement	0 (0)	2 (0.9)	0.499
Pathological TNM stage			0.957
0	50 (21.8)	52 (22.7)	
I	59 (25.8)	63 (27.5)	
II	58 (25.3)	57 (24.9)	
III	60 (26.2)	58 (24.5)	
IV	2 (0.9)	1 (0.4)	
TRG grade			**0.026**
0	51 (21.8)	52 (22.7)	1.000
1	84 (36.7)	61 (26.6)	**0.027**
2	77 (33.6)	105 (45.9)	**0.010**
3	17 (7.4)	11 (4.8)	0.330
Perineural invasion	11 (4.8)	12 (5.2)	1.000
Vascular invasion	11 (4.8)	9 (3.9)	0.820

* *Some patients experienced more than one complication, and categorized as. NCRT, neoadjuvant chemoradiotherapy; NCRT-Cape, neoadjuvant chemoradiotherapy and additional capecitabine chemotherapy; CRM, circumferential resection margin; TRG, tumor regression grade. Bold values indicate of statistical significance*.

### Pathological Outcomes

**Figure 2:**
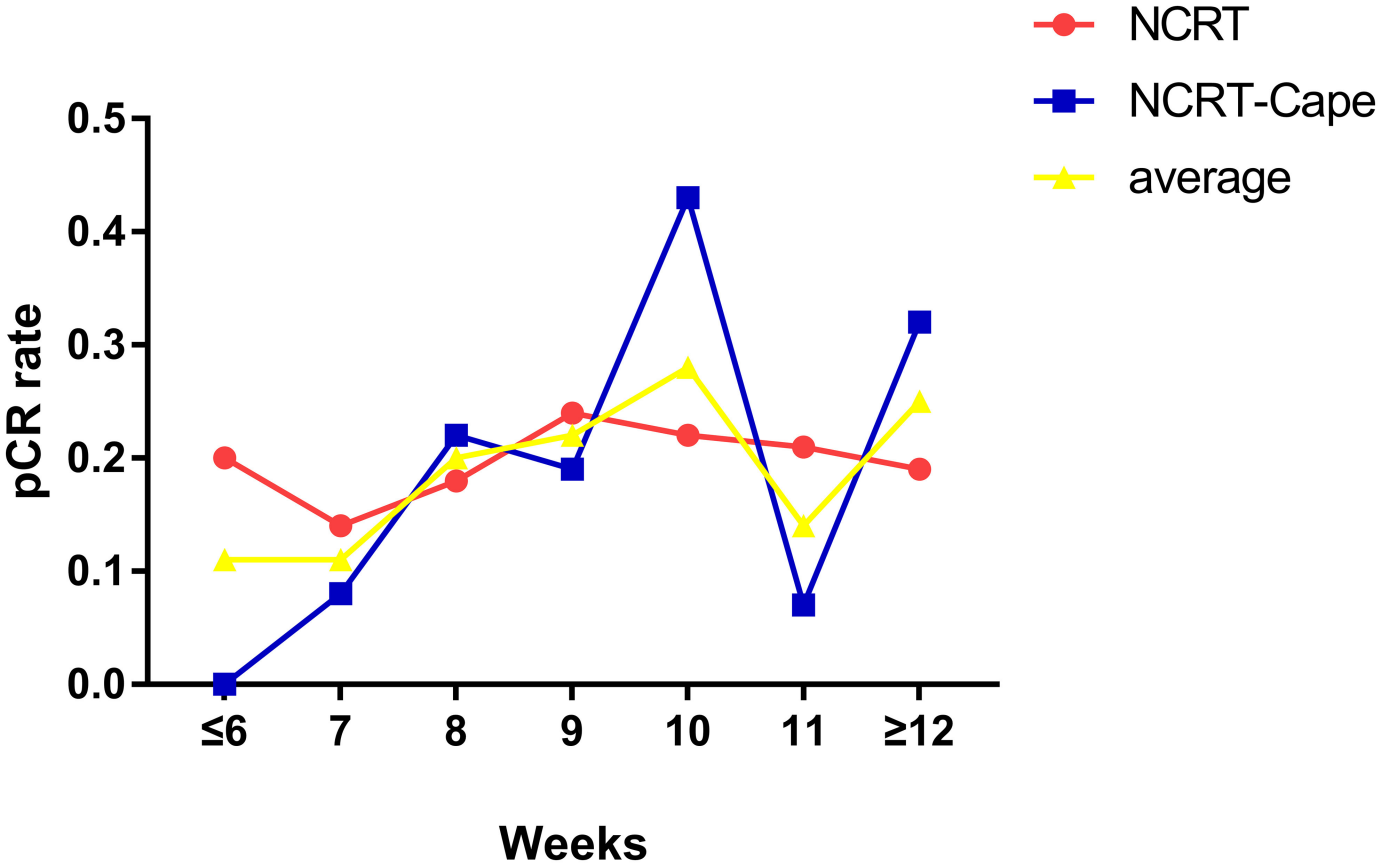
The relationship between time intervals and pCR rates. NCRT, neoadjuvant chemoradiotherapy; NCRT-Cape, neoadjuvant chemoradiotherapy, and additional capecitabine chemotherapy.

Adding consolidation capecitabine chemotherapy had no impact on lymph node retrieved and metastatic lymph nodes (*p* = 0.832, *p* = 0.840, respectively). With regard to tumor response to NCRT, a lower proportion of good response (TRG1: 36.7 vs. 26.6%, *p* = 0.028) and a higher proportion of partial response (TRG2: 33.6 vs. 45.9%, *p* = 0.010) were noted in patients in the NCRT-Cape group. However, additional administration of 2 cycles of consolidation capecitabine chemotherapy did not increase pCR rate compared to standard NCRT group (21.8 vs. 22.7%, *p* = 1.000, Figure 2). Positive circumferential resection margin rates were comparable between both groups, and tumor size (*p* = 0.499, *p* = 0.485). A pathological TNM stage was similar between two groups (*p* = 0.957). Similarly, perinerval and vascular invasion did not differ between two groups (*p* = 1.000, *p* = 0.820, respectively).

### Predictive Factors of pCR

To identify risk factors for pCR in LARC, logistic regression analysis was performed in 545 patients (before propensity score matching). In univariate analysis, adding consolidation capecitabine chemotherapy (OR = 0.954, *p* = 0.476) was not correlated with pCR in patients with LARC. Tumor size (OR = 0.428, *p* < 0.001), interval time between NCRT and surgery (OR = 1.141, *p* = 0.036), pre-NCRT clinical T stage (OR = 0.641, *p* = 0.027), pre-NCRT clinical N stage (OR = 0.514, *p* = 0.031), lymph nodes harvested (OR = 0.955, *p* = 0.008), and post-NCRT carcinoembryonic antigen (CEA) (OR = 0.872, *p* = 0.003) were significantly correlated with pCR in patients with LARC. On multivariate analysis, tumor size (OR = 0.439, *p* < 0.001), and interval time between NCRT and surgery (OR = 1.241, *p* = 0.003), and post-NCRT CEA (OR = 0.880, *p* = 0.008) were significant risk factors for pCR in patients with LARC (Table 3).

**Table 3:** Univariate and multivariate analysis of predictive factors for pCR in locally advanced rectal cancer patients (*n* = 545).

**Variables**	**Univariate analysis**			**Multivariate analysis**		
	**HR**	**95% CI**	***p*** **-Value**	**HR**	**95% CI**	***p*** **-Value**
Sex, male/female	1.383	0.908–2.105	0.926			
Age	0.999	0.980–1.017	0.886			
ASA	0.916	0.594–1.411	0.689			
Distance from the anal verge	0.927	0.850–1.011	0.088			
Tumor size	0.428	0.334–0.548	**<0.001**	0.439	0.338–0.570	**<0.001**
Surgery approach						
Laparoscopic	Reference	Reference	0.724			
Open	1.141	0.530–2.455	0.736			
Robotic	1.348	0.582–3.122	0.486			
Sphincter-Saving procedure	0.584	0.279–1.221	0.153			
Operative time (min)	0.998	0.995–1.002	0.337			
Estimated blood loss (ml)	0.998	0.995–1.001	0.284			
Time interval between NCRT and surgery	1.141	1.009–1.291	**0.036**	1.241	1.074–1.434	**0.003**
Pre-NCRT cT stage	0.641	0.432–0.950	**0.027**	0.710	0.460–1.096	0.123
Pre-NCRT cN stage	0.514	0.281–0.941	**0.031**	0.707	0.355–1.407	0.323
Post-Operative hospital stay	0.969	0.927–1.014	0.175			
Lymph nodes harvested	0.955	0.924–0.988	**0.008**	0.985	0.948–1.022	0.420
Post-NCRT CEA level	0.872	0.795–0.956	**0.003**	0.880	0.802–0.967	**0.008**
Post-NCRT CA19-9 level	0.993	0.979–1.007	0.309			
Post-Operative complications	0.821	0.456–1.475	0.666			
Plus capecitabine	0.954	0.632–1.440	0.822			
Reduce the dose	1.237	0.246–6.210	0.796			
NCRT complications	1.139	0.721–1.800	0.577			

*NCRT, neoadjuvant chemoradiotherapy; NCRT-Cape, neoadjuvant chemoradiotherapy, and additional capecitabine chemotherapy; CRM, circumferential resection margin; TRG, tumor regression grade, HR, hazard ratio; CI, confidential interval. Bold values indicate of statistical significance*.

### Nomogram for pCR

**Figure 3:**
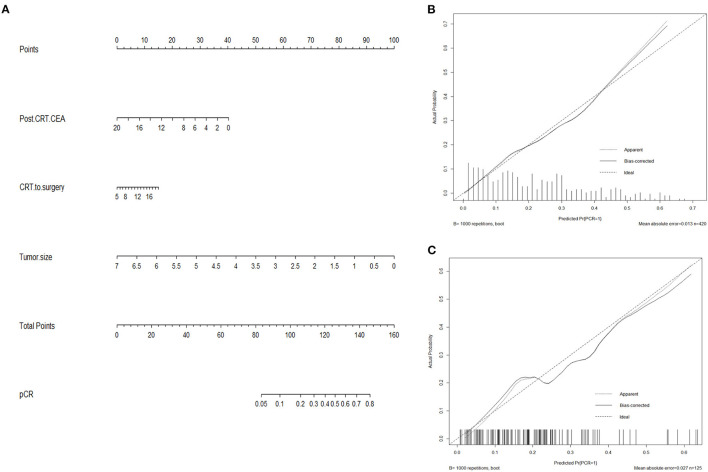
Nomogram predicting pCR **(A)** and calibration curves with internal **(B)** and external **(C)** validation. **(A)** A score for each variable can be obtained at the top scale, and the sum of scores indicates a predictive probability of pCR. **(B,C)** The solid line indicates the actual performance of our nomogram, and the dashed line represents the prediction by an ideal model. CEA, carcinoembryonic antigen.

Based on results from multivariate analysis, a predicting nomogram for pCR was developed, as demonstrated in Figure 3A. By summing up the score of each variable, a straight line could be drawn to obtain the predicted pCR rate. The C-index of the nomogram was 0.78 (95% CI 0.73–0.83). The calibration curve (Figure 3B) showed good performance upon internal validation between the predicted and actual probability of pCR. Upon external validation, the C-index of the nomogram was 0.73 (95% CI 0.63–0.83), and the calibration curve (Figure 3C) showed good accordance between predicted and observed probabilities of pCR.

## Discussion

A great effort has been made to maximize tumor response to NCRT, in which pCR is selected as a surrogate endpoint. Herein, we investigated the efficacy of adding consolidation capecitabine chemotherapy without changing the CRT-to-surgery interval for patients with LARC. The result demonstrated that adding 2 cycles of consolidation capecitabine to NCRT had no impact on the short-term perioperative outcome, and did not result in an increase in pCR rates. Additionally, by incorporating post-NCRT significant predictive factors in Logistic analysis, we built a nomogram for pCR that might assist in decision-making about organ-preserving strategies.

Fluoropyrimidine-based (5-FU or capecitabine) pre-operative chemoradiotherapy is the standard care for LARC. Incorporation of oxaliplatin to fluoropyrimidine-based CRT has been shown to acquire equivalent oncological outcomes but increase toxicities (18–22). Accumulating evidence has proposed capecitabine to be an alternative to 5-FU, the efficacy of XELOX (capecitabine and oxaliplatin) was comparable with that of the FOLFOX4 regimen (5-FU/folinic acid plus oxaliplatin) (26). In addition, using oral capecitabine instead of infusional 5-FU during NCRT has been shown to be correlated with improved tumor response and lower toxicity, and a comparable pCR rate is compared to pre-operative 5-FU-based NCRT (27–29). It has been demonstrated that nCRT with capecitabine is safe and well-tolerated in the ACCORD/PRODIGD 2 phase III trial (30).

To improve tumor response without increasing perioperative complications, we added 2 cycles of consolidation capecitabine chemotherapy in the waiting period to the standard NCRT regimen. Our preliminary results demonstrated that additional two-cycle consolidation capecitabine did not increase the incidence of peri-CRT complications, such as hand-foot syndrome, fatigue, and diarrhea, suggesting this regimen might be well-tolerated.

Meanwhile, we used propensity score matching to reduce selection bias; between-group baseline characteristics were well-balanced after matching. No negative effect on surgical outcome was observed when patients were administered two-cycle consolidation capecitabine to NCRT. Regarding surgical morbidity, the severity of post-operative complications and post-operative hospital stay were found comparable between the two groups. No reoperation and perioperative mortality occurred in the two groups. Together, these results indicated the safety of adding 2 cycles of consolidation capecitabine to the NCRT regimen.

To further explore the efficacy of adding consolidation capecitabine to NCRT on tumor response, we compared the pCR rates between the two groups. The addition of consolidation capecitabine to NCRT did not significantly improve pCR rate. The absolute ypCR rate difference between groups was very small and not statistically significant. Similar results were found in a recent phase II OIGIT-01 Trial (31). We suggested that radiosensitization by using capecitabine might have already maximized tumor responses to NCRT, leaving little room for improvement with the addition of two-cycle consolidation capecitabine chemotherapy.

The optimal timing of surgery after NCRT is still controversial (32). Surgery beyond 8 weeks after completion of radiotherapy might increase pelvic fibrosis and rectal edema, leading to intraoperative technical difficulties and increased surgical complications. On the other hand, the pCR rate might increase by prolongation of the NCRT-to-surgery interval (17, 33–36). Herein, we analyzed the relationship between pCR and NCRT-to-surgery interval. The result revealed that longer interval time correlated with increased pCR rate. Results from Logistic regression demonstrated that interval time was a significant risk factor for pCR than the consolidation capecitabine chemotherapy.

Additionally, after adjustment for confounding factors, tumor size, and post-NCRT CEA level were significant risk factors for pCR in patients with LARC. To facilitate the decision-making regarding organ-preserving strategies, we developed a nomogram predicting pCR. This nomogram has a reliable C-index on internal and external validation. The incorporation of specific molecular and genetic markers into the predicting nomogram would enhance the performance of the model. Nowadays, delivery of “total neoadjuvant therapy” (TNT) strategies is becoming increasingly popular to improve the pCR rates (37), allowing a group of patients to benefit from full-dose adjuvant chemotherapy and finally a less-invasive organ preservation strategy (38–41). However, the definite role of TNT strategies is still unveiled.

There are several limitations that warrant discussion. First, this study was subjected to selection bias owing to its retrospective nature. To minimize selection bias between groups, we performed propensity score analysis. Second, our predictive model was based on a single-center retrospective analysis. It requires further external validation in a large population from multiple institutions. Another limitation was that this study focused on the pCR rate, a surrogate marker of oncological outcomes. Further studies focused on the long-term oncological outcomes are needed to further confirm the results of our study. Nevertheless, our study adds to the understanding of the efficacy of adding capecitabine to standard NCRT.

Our study suggested that additional studies in a large-scale population are needed to confirm these results.

## Data Availability Statement

The original contributions presented in the study are included in the article/Supplementary Material, further inquiries can be directed to the corresponding author/s.

## Ethics Statement

Studies relative to humans in this article were approved by the Ethics Committee of The Fujian Medical University Union Hospital (2013051). The patients/participants provided their written informed consent to participate in this study.

## Author Contributions

YFF, CMS, FD, WJZ, GXG, and XL designed the experiments, performed the experiments, analyzed the data, and wrote the article. All authors contributed to the article and approved the submitted version.

## Funding

This study was supported by the National Foundation of China (No. 82172800), Science Foundation of the Fujian Province (No. 2019J0105), Special Financial Foundation of Fujian Provincial (No. 2015-1297 and 2020B1050), the Startup Fund for Scientific Research, Fujian Medical University (2017XQ1029 and 2018QH2027), and the Professor Development Foundation of Fujian Medical University (No. JS11006). Talent programs granted from The First Affiliated Hospital of Fujian Medical University (YJRC3600). Joint Funds for the Innovation of Science and Technology, Fujian Province (2020Y9125).

## Conflict of Interest

The authors declare that the research was conducted in the absence of any commercial or financial relationships that could be construed as a potential conflict of interest.

## Publisher's Note

All claims expressed in this article are solely those of the authors and do not necessarily represent those of their affiliated organizations, or those of the publisher, the editors and the reviewers. Any product that may be evaluated in this article, or claim that may be made by its manufacturer, is not guaranteed or endorsed by the publisher.
